# Flexocatalytic Hydrogen Generation and Organics Degradation by Nano SrTiO_3_

**DOI:** 10.1002/advs.202500034

**Published:** 2025-04-25

**Authors:** Susmita Mondal, Rajib Chandra Das, Yumeng Du, Zhenyuan Hou, Konstantin Konstantinov, Zhenxiang Cheng

**Affiliations:** ^1^ Institute for Superconducting and Electronic Materials Faculty of Engineering and Information Sciences University of Wollongong Squires Way North Wollongong NSW 2500 Australia

**Keywords:** Flexocatalysis, Hydrogen generation, Mechanocatalysis, Renewable energy, Strontium Titanate

## Abstract

Flexocatalysis, a groundbreaking approach in mechanocatalysis, overcomes the material limitations imposed by the symmetry requirements of piezocatalysis, enabling a broader range of materials to generate free radicals through mechano‐catalytic reactions. This method not only offers an eco‐friendly pathway for green hydrogen production via water splitting but also facilitates the use of biocompatible materials in health diagnostics and treatments. In this study, the flexocatalytic activity of centrosymmetric SrTiO_3_ (STO) nanopowders is demonstrated, achieving notable hydrogen evolution (1289.53 µmol g^−1^ h^−1^ in pure water) and efficient organic dye degradation (≈94%). The mechanism is driven by electric polarization generated under non‐uniform strain, which is strongly enhanced with particle size reduction, effectively linking flexoelectricity to superior electrochemical performance. The findings highlight the potential of flexocatalysis to revolutionize hydrogen production and broaden the range of materials for catalytic applications, paving the way for innovative energy‐harvesting technologies.

## Graphical abstract

Flexocatalysis, driven by non‐uniform strain‐induced electric polarization, overcomes piezocatalysis limitations, enabling versatile mechano‐catalytic applications. This study highlights the flexocatalytic activity of STO nanopowders, achieving significant hydrogen evolution (1289.53 µmol g^−1^ h^−1^) and ≈94% dye degradation. The findings establish a strong link between flexoelectricity and enhanced electrochemical performance, offering eco‐friendly solutions for hydrogen production and catalytic innovation.

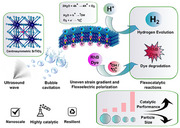

## 1 Introduction

The global demand for clean, sustainable energy, compounded by the depletion of fossil fuel reserves and the urgent need to reduce environmental damage, underscores the necessity for innovative energy solutions. Among the most promising is hydrogen production via water splitting. Hydrogen, with its high energy density, abundance, and low environmental impact, presents a compelling solution to both energy and ecological challenges.^[^ ^1^, ^2^ ^]^ However, unlocking hydrogen's full potential requires the development of water‐splitting technologies that are not only efficient but also cost‐effective and environmentally sustainable.^[^ ^3^ ^]^ One innovative approach addressing this challenge is piezocatalysis, which leverages the unique properties of piezoelectric materials to drive chemical reactions, including hydrogen generation, through mechanical stimuli. In piezocatalysis, mechanical stress—such as ultrasound vibrations—induces polarization within piezoelectric materials, generating an internal electric field. This field promotes the separation and migration of charge carriers (electron‐hole pairs) to the material's surface, facilitating catalytic reactions.^[^ ^4^, ^5^ ^]^ Despite its potential, piezocatalysis is limited to non‐centrosymmetric materials, which restricts the range of suitable materials. Moreover, concerns over the environmental safety and biocompatibility of certain piezoelectric materials constrain their broader application, particularly in green energy and biomedical technologies.^[^ ^6^ ^]^

Recent advances in material science have introduced flexoelectricity as a new paradigm capable of overcoming the structural limitations of piezocatalysis. Unlike piezoelectricity, flexoelectricity can occur in any material, regardless of its symmetry, by generating electrical polarization in response to non‐uniform mechanical strain gradients.^[^ ^7^ ^]^ At the nanoscale, where strain gradients are more pronounced, flexoelectric polarization can mimic piezoelectric effects.^[^ ^8^, ^9^ ^]^ Methods such as mechanical bending, strain relaxation, and ultrasonic vibrations can induce significant flexoelectric responses, offering a promising mechanism for energy conversion and catalysis.^[^ ^10^, ^11^, ^12^, ^13^, ^14^ ^]^ The combination of ultrasonic oscillations and flexoelectricity opens new avenues for catalysis that extend beyond the limitations of piezoelectric materials. This application of flexoelectricity in catalysis, termed flexocatalysis, has demonstrated considerable potential. For instance, 2D MnO_2_ has shown effective flexocatalytic activity in the degradation of organic pollutants under ultrasonic stimulation.^[^ ^15^ ^]^ Similarly, rutile‐phase TiO_2_ has exhibited outstanding performance in both hydrogen evolution and dye degradation, confirming the viability of flexoelectric mechanisms in catalytic processes.^[^ ^16^ ^]^ These promising results have fueled interest in discovering new materials that can enhance flexocatalytic efficiency and broaden the scope of applications, from environmental remediation to sustainable energy generation.

Among these candidates, STO, a well‐known centrosymmetric perovskite, stands out as a particularly promising material for flexocatalysis. STO has been extensively studied as a photocatalyst for water splitting and organic pollutant degradation due to its favorable band positions and bandgap (≈3.25 eV).^[^ ^17^, ^18^, ^19^ ^]^ While bandgap can be altered by the process conditions,^[^ ^20^, ^21^, ^22^ ^]^ it plays crucial role on the value and quantification of redox potential, important for catalytic reaction.^[^ ^23^ ^]^ Furthermore, STO possesses desirable properties, including a high dielectric constant, strong thermal stability, and tunable electrical characteristics.^[^ ^24^, ^25^, ^26^, ^27^, ^28^ ^]^ These features make STO an ideal platform for exploring flexoelectric effects in catalytic applications. By inducing flexoelectric polarization in STO, it is possible to unlock catalytic functionalities otherwise restricted by its centrosymmetric structure.

In this study, we investigate the flexocatalytic properties of STO nanopowder under ultrasonic vibrations, focusing on its performance in hydrogen evolution and organic dye degradation. Our findings reveal that STO exhibits impressive catalytic activity, achieving hydrogen evolution rates of 1289.53 µmol g⁻¹ h⁻¹ in pure water and 94% degradation efficiency for organic dyes. These results surpass those achieved by many known piezoelectric materials, highlighting the significant potential of flexocatalysis for sustainable energy conversion and environmental remediation. Moreover, our computational simulations highlight the critical impact of particle size on enhancing flexoelectric polarization, with predicted values ≈ 48.02 nC m^−^^2^, emphasizing the importance of nanoscale engineering in optimizing catalytic performance. The results further support the superior catalytic activity of pure STO (STO‐P), attributed to its smaller particle size (35 nm), as confirmed by TEM analysis, compared to the other two STO samples (STO‐3 and STO‐24, annealed in air at 1000 °C for 3 h and 24 h respectively). These findings not only expand the range of materials suitable for catalytic applications via flexoelectric mechanisms but also introduce innovative strategies for sustainable hydrogen production. STO's scalability and environmental compatibility underscore the transformative potential of flexocatalysis in advancing clean energy technologies.

## 2 Results and Discussion

### 2.1 Structural Characterization

**Figure 1:**
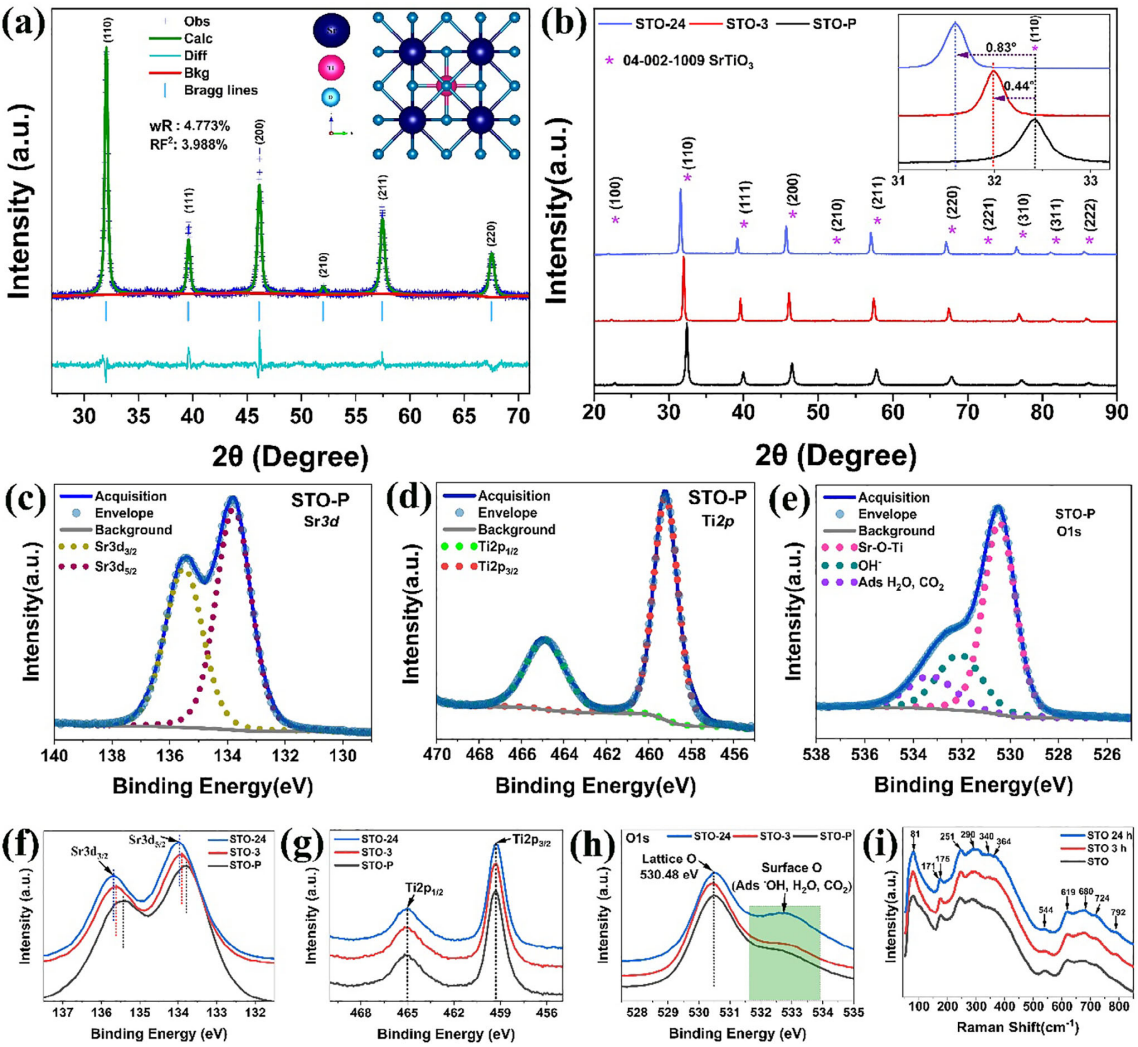
Structural and chemical analysis of STO nanopowders. a) XRD patterns of STO nanopowders with Rietveld refinement results and the unit cell (inset) on the right. (wR: data residual; RF^2^: unweighted phase residuals). b) Relative XRD peak shift of heat‐treated STO samples compared to untreated STO and the reference PDF_96‐721‐2246. c–e) Deconvoluted XPS spectra of STO‐P, presenting the individual components of the Sr 3d, Ti 2p, and O 1s peaks. f–h) Comparative XPS peak positions for Sr, Ti 2p, and O 1s states in STO‐P, STO‐3, and STO‐24 samples. The surface oxygen peak is highlighted in green. i) Raman spectra of STO‐P, STO‐3, and STO‐24, illustrating the evolution of vibrational modes with heat treatment.

The X‐ray diffraction (XRD) patterns of the STO‐P nano‐powder, along with the Rietveld refinement results, are depicted in **Figure** **1**a. The inset displays the structural model of the unit cell for the refined phase of STO‐P. Rietveld refinement was conducted to attain the optimal refinement profile and values of reliability factors (wR and RF^2^), which are crucial for assessing sample purity. The profile plot demonstrates a commendable fit, with diffraction peaks indexed via Bragg reflections, affirming that the sample has single‐phase nature, adhering to the standard pattern of cubic STO with the space group of Pm̅3 m, 221 (PDF# 04‐002‐1009, a = 3.9050 Å). The refined structure of STO, visualized using Vesta 3 software (Figure S1, Supporting Information), reveals a centrosymmetric arrangement. Strontium atoms occupy the corners, while titanium atoms reside at the center of the perovskite unit cell. The structure comprises SrO_12_ cuboctahedra and TiO_6_ octahedra, with a balanced charge distribution.

Figure 1b illustrates the XRD peaks of STO‐P and heat‐treated STO‐3 and STO‐24, showing their relative peak positions compared to the standard STO peak, marked by an asterisk (*). A significant transformation is observed in the heat‐treated STO samples: a pronounced leftward shift of the intense XRD peak (110), with no other alterations detected, as shown in the inset. This shift, measuring 0.44° after 3 h and 0.83° after 24 h at 1000 °C, indicates lattice relaxation due to crystal growth while maintaining the cubic phase. These findings highlight the crucial role of both temperature and extended sintering duration in promoting crystal development within STO nanopowder specimens.^[^ ^29^ ^]^

Figure 1c–e represents the X‐ray photoelectron spectroscopy (XPS) spectra of STO‐P representing the fitted Sr 3d, Ti 2p, and O 1s states respectively. Sr 3d spectrum (Figure 1c) shows a doublet peak corresponding to spin‐orbit split components at 133.78 eV and 135.48 eV, respectively, which correspond to the Sr 3d_3/2_ and Sr 3d_5/2_ states of Sr^2+^ ions in the STO nanopowder.^[^ ^30^, ^31^ ^]^ In the Ti 2p spectra (Figure 1d), the two distinctive peaks for Ti 2p_1/2_ and Ti 2p_3/2_ at 464.88 eV and 459.28 eV respectively can be attributed to Ti^4+^ in STO.^[^ ^32^ ^]^ The O1s spectrum (Figure 1e) is fitted into three different peaks. Peak at 529 eV is associated with lattice oxygen. Whereas peak at 532 eV and 533 eV are responsible for surface adsorbed hydrox ^•^OH and H_2_O respectively.^[^ ^33^ ^]^ XPS analysis was conducted on STO‐3 and STO‐24 to assess potential shifts in their chemical states compared to the reference STO‐P sample. The fitted XPS spectra for the Sr 3d, Ti 2p, and O 1s states of both materials are shown in Figure S2 (Supporting Information). Furthermore, Figure 1f–h illustrates the relative peak positions of Sr 3d, Ti 2p, and O 1s states in heat‐treated STO compared to pure STO samples. Interestingly, the Sr 3d_3/2_ and Sr 3d_5/2_ states exhibited a slight shift to higher energy after treatment at 1000 °C, with this shift becoming more pronounced with extended duration at the same temperature (Figure 1f). This shift in the Sr core level is likely due to increased strain from crystal growth at high temperatures. Crucially, this shift is not associated with any phase change, as evidenced by the stable peak positions of Ti 2p (Figure 1g) and lattice oxygen at 530.48 eV (Figure 1h). However, a noticeable change is observed in the O1s peak for STO‐24 samples, highlighted in the green box in Figure 1h, indicating alterations in surface oxygen content due to prolonged heat treatment. Overall, the XPS analysis confirms that no phase change occurred, despite the observed shifts in Sr peaks and changes in surface oxygen content for STO‐24.

Raman spectroscopy was conducted at room temperature to investigate the phase and any transitions of STO after heat treatment. The broad and weaker Raman peaks shown in Figure 1i indicate second‐order modes, which dominate the 200–400 cm⁻¹ and 500–800 cm⁻¹ ranges. First‐order modes are absent, as they are forbidden for the centrosymmetric perovskite structure of cubic STO at room temperature.^[^ ^34^ ^]^ The most notable peaks include a combination of transverse optical modes and transverse acoustic modes at the 81 cm⁻¹ range (TO1‐TA and/or TO2‐TO1 combination band), TO2 at 175 cm⁻¹, and longitudinal optical modes LO1 at 171 cm⁻^1^.^[^ ^34^, ^35^ ^]^ The 251 cm⁻¹ band represents overtones (2TA and 2TO1) and the combination TO1+TA.^[^ ^34^ ^]^ All three STO samples exhibited similar Raman peaks, except for the LO4 mode at 792 cm⁻¹,^[^ ^35^ ^]^ which appeared in samples heated to 1000 °C for both 3 and 24 h. This is attributed to increased stress on the material at high temperatures rather than a phase change.

The Brunauer‐Emmett‐Teller (BET) surface areas and pore parameters of the samples were tested and analyzed by N_2_ gas adsorption/desorption method. The isotherms of all the samples were identified as a combination of types IV and are presented in supporting file (Figure S3, Supporting Information). Distinct H3 hysteresis loops were observed in the range of 0.0–1.0 P/P_0_, indicating that these samples had a porous structure. The surface area decreased with increasing annealing temperature, likely due to particle growth in the annealed samples. STO‐P exhibited the highest surface area of 22.89 ± 0.06 m^2^ g^−1^, which reduced to 8.44 ± 0.08 m^2^ g^−1^ and 7.84 ± 0.05 m^2^ g^−1^ for STO‐3 and STO‐24, respectively.

**Figure 2:**
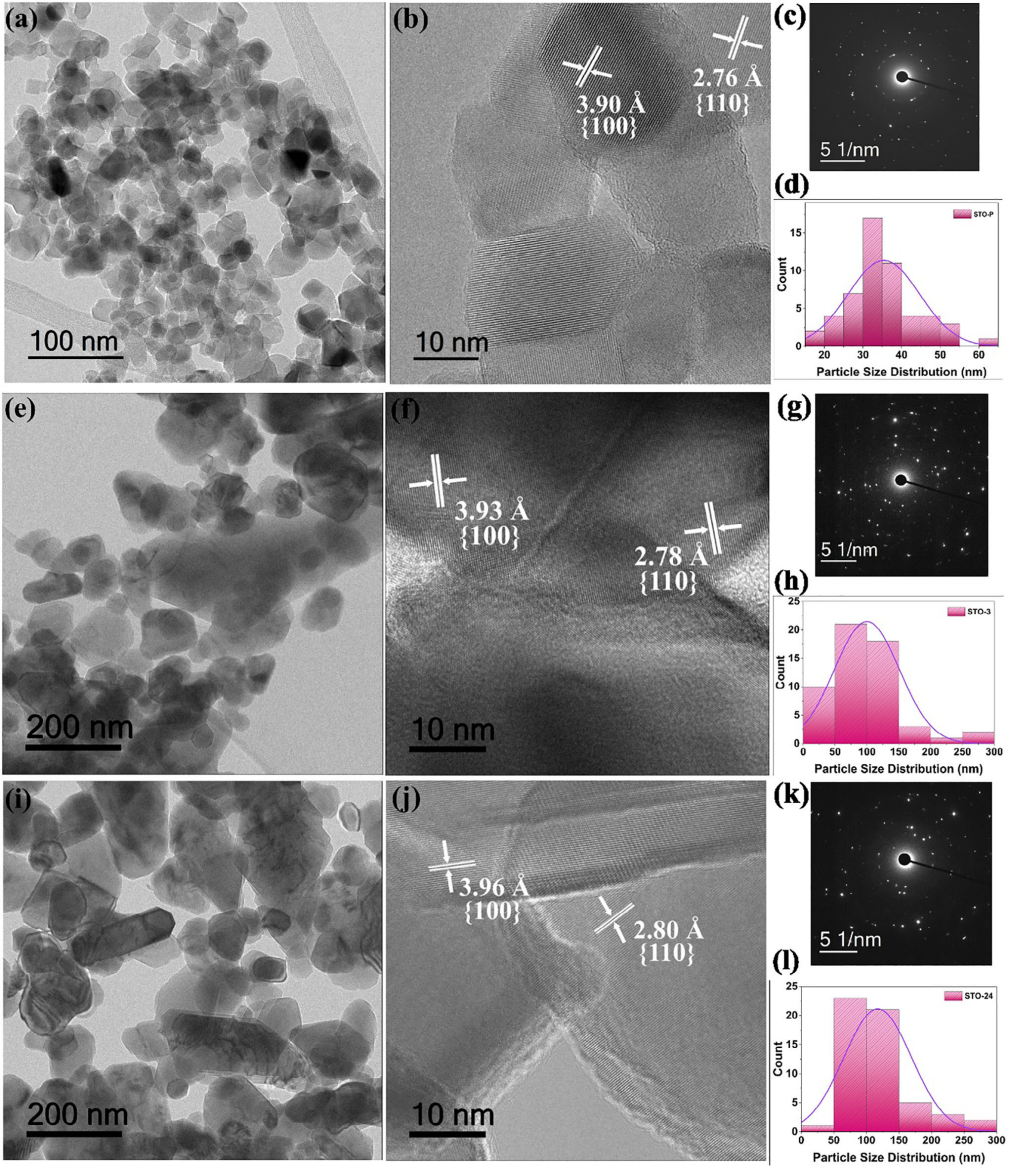
Morphological and crystallographic characterization of STO nanopowders. a) TEM image of STO‐P nanopowder. b) HRTEM showing lattice fringes with d‐spacing values of STO‐P. c) SAED pattern of pure STO‐P. d) Particle size distribution of STO‐P obtained from TEM image. e) TEM image of STO‐3. f) HRTEM showing lattice fringes with d‐spacing values of STO‐3. g) SAED pattern of STO‐3. h) Particle size distribution of STO‐3 obtained from TEM image. i) TEM image of STO‐24. j) HRTEM showing lattice fringes with d‐spacing values of STO‐24. k) SAED pattern of STO‐24. l) Particle size distribution of STO‐24 obtained from TEM image.

Transmission electron microscopy (TEM) and high resolution TEM (HRTEM) images provided deeper insights into the lattice structure and morphology of the STO samples. As shown in **Figure** **2**a,e,i, the TEM images of STO‐P, STO‐3, and STO‐24 samples reveal that the particles grew significantly upon annealing at high temperatures, with average particle sizes of 35 nm, 100 nm, and 117 nm, respectively. The STO‐P nanoparticles exhibit a regular, consistent rectangular shape in the TEM images. However, as the temperature and holding duration increased in the STO‐3 and STO‐24 samples, these rectangular particles became more irregular, indicating changes in their morphology.

HRTEM images revealed the d‐spacing values for the STO samples, providing key insights into their crystal structures. For STO‐P, the d‐spacing values of 3.90 Å and 2.76 Å correspond to the (100) and (110) planes. Similarly, STO‐3 showed d‐spacing values of 3.93 Å and 2.78 Å for the same planes. Meanwhile, STO‐24 exhibited d‐spacing values of 3.96 Å and 2.80 Å, again corresponding to the (100) and (110) planes. These HRTEM results are consistent with those obtained from XRD analysis, confirming the reliability of the structural information derived from both techniques. These findings confirm that all STO samples maintain the same crystal phase, with heat treatment not causing any phase shifts. Additionally, the polycrystalline nature of the samples was validated through the selected area electron diffraction (SAED) patterns.

### 2.2 Catalytic Performance of STO

**Figure 3:**
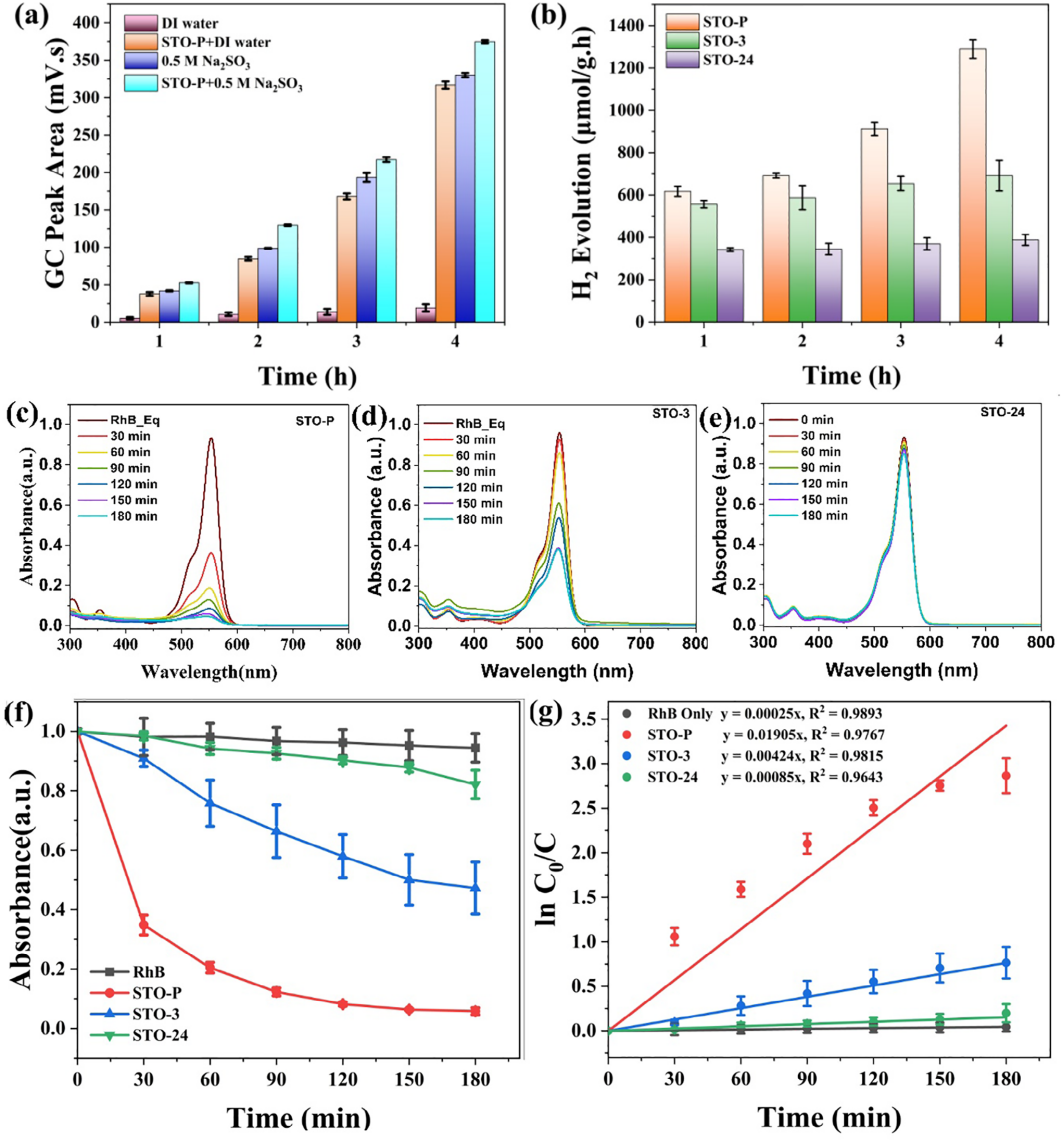
Flexocatalytic hydrogen evolution and dye degradation under ultrasonic vibration. a) Comparison of hydrogen evolution performance with and without catalysts in DI water and 0.05 M Na_2_SO_3_ over 4 h intervals, logging peak area as a variable. b) Hydrogen evolution rate comparison between pure and heat‐treated STO samples. c–e) Flexocatalytic performance of organic dye (RhB) degradation using STO‐P, STO‐3, and STO‐24 with insets showing RhB concentration (C_o_ = 5 mg L^−1^) over time. f) Relative dye degradation performance of STO‐P, and heat‐treated STO samples. g) ln (C_o_/C) versus vibration time plots, fitted to pseudo‐first‐order kinetics.

**Figure** **3** illustrates the catalytic performance of STO in hydrogen evolution from water and the degradation of the organic dye RhB under ultrasonic vibration (200 W, 40 kHz) in dark conditions. Figure 3a depicts hydrogen generation from deionized (DI) water alone and DI water with a sacrificial agent sodium sulphite (0.05 M Na_2_SO_3_) both in the presence and absence of the STO catalyst. A small amount of hydrogen (0.17 µmol h^−1^) was produced from DI water alone after 1hr sonication, and this amount steadily increased over the subsequent 4 h. This increase is primarily attributed to the breakdown of water molecules under ultrasonic vibrations, leading to the formation of H · and · OH radicals. The combination of two H · radicals results in the formation of H_2_.^[^ ^36^, ^37^ ^]^ Previous studies have demonstrated that water splitting can occur at an early stage without additional chemicals, as sonolysis generates free radicals under appropriate sonication conditions.^[^ ^38^, ^39^ ^]^

The introduction of the STO catalyst significantly enhanced hydrogen production. Over 4 h, average yields reached 1525.30 µmol g^−1^ h^−1^ in DI water with 0.05 m Na_2_SO_3_ and 1289.53 µmol g^−1^ h^−1^ in DI water alone. When compared to control experiments (hydrogen generation from DI water alone and Na_2_SO_3_ alone, as shown by relative GC peak area), the addition of STO clearly resulted in a substantial increase in hydrogen generation (Figure 3a). This enhancement is attributed to the flexocatalytic properties of STO. The flexocatalytic mechanism for hydrogen generation can be described by the following Equations ((1), (2), (3)):

(1) $$\mathrm{SrTi}O_{3}\overset{\mathrm{Ultrasound}}{\to }\mathrm{SrTi}O_{3}+e^{-}+h^{+}$$

(2) $$2h^{+}+H_{2}O\to 2H^{+}+\frac{1}{2}O_{2}$$

(3) $$2H^{+}+2e^{-}\to H_{2}$$

STO nanoparticles generate positive (holes, h^+^) and negative (electrons, e^−^) charges on the reactive sites of their surfaces under ultrasonic vibrations (Equation 1). This is due to the flexoelectric effect, which induces a strain gradient through bubble cavitation.^[^ ^40^ ^]^ The positive charges react with water molecules, producing H^+^ and O_2_ (Equation 2), while the negative charges quench hydrogen ions, forming H_2_ (Equation 3).^[^ ^41^ ^]^ Oxygen vacancies on the STO surface serve as reactive sites and are influenced by particle size and surface area. Smaller particles have a higher density of oxygen vacancies due to their larger surface area to volume ratio. Some studies modify the material surface to increase oxygen vacancies and enhance catalytic activity.^[^ ^42^, ^43^ ^]^ XPS analysis (O1s scan, Figure 1h) showed the presence of adsorbed · OH on the surface of all three STO materials. This suggests the presence of oxygen vacancies, as hydroxylation tends to occur at oxygen‐deficient sites.^[^ ^44^, ^45^ ^]^ Furthermore, TEM and BET surface area analysis showed that STO‐P has approximately three times smaller particle size and higher surface area than STO‐3 and STO‐24. This suggests that STO‐P has more active sites due to a higher concentration of oxygen vacancies, resulting in its superior catalytic activity compared to the both heat‐treated STO samples.

It is noteworthy that a significant amount of hydrogen was produced from DI water with 0.05 m Na_2_SO_3_, even without the STO catalyst, as evidenced by the large GC peak in Figure 3a. Na_2_SO_3_, in general acts as a sacrificial agent, capture h^+^ generated during catalytic system (as shown in Equation 4), preventing their recombination with e. This ensures a continuous supply of electrons for proton reduction and subsequent hydrogen production.

However, in this study under ultrasonication, Na_2_SO_3_ also directly reacts with water to generate hydrogen, in addition to its established hole‐scavenging mechanism. In the presence of STO, which generates an abundance of holes under ultrasonication, Na_2_SO_3_ primarily functions as a hole scavenger. However, in the absence of STO, a limited number of holes are still generated under continuous sonication. Upon dissolution in water, Na_2_SO_3_ forms a weakly alkaline solution with a pH of ≈8.50 at room temperature (22.5 °C) due to hydrolysis (Equation 4), which increases to 8.75 after reacting with H_2_O, this increase in pH indicate a rise in OH⁻ concentration. This suggests that the reversible reaction in (Equation 5) shifts toward the forward direction. Furthermore, the $HSO_{3}^{-}$ formed in (Equation 5) undergoes subsequent decomposition (Equation 6), leading to the generation of H⁺, which ultimately contributes to hydrogen evolution (Equation 7). This hypothesis aligns with recent findings demonstrating hydrogen generation from Na_2_SO_3_.^[^ ^46^ ^]^

Since the estimated top valence band level of STO‐P (≈ 2.25 eV vs NHE) is significantly more positive than the reduction potential of ${\mathrm{SO}}_{3}^{•-}$) (≈ 0.75 eV vs NHE), Na_2_SO_3_ proves to be well‐suited for this application. In the presence of STO during catalysis, the solution pH also slightly increases to ≈8.70. However, in this case, the pH rise is attributed to the consumption of hydrogen ions during hydrogen generation.^[^ ^16^ ^]^

(4) $${\mathrm{SO}}_{3}^{2-}+h^{+}\to {\mathrm{SO}}_{3}^{•-}$$

(5) $${\mathrm{SO}}_{3}^{2-}+H_{2}O⇌{\mathrm{HSO}}_{3}^{-}+OH^{-}$$

(6) $$3{\mathrm{HSO}}_{3}^{-}\to S^{\circ }+2{\mathrm{SO}}_{4}^{2-}+H_{2}O+H^{+}$$

(7) $$2H^{+}+2e^{-}\to H_{2}$$

While sacrificial agents enhance hydrogen evolution, they also introduce complexities such as by‐product formation, purification challenges, and increased costs, making this approach less practical. Notably, hydrogen production using the STO catalyst without a sacrificial agent is competitive with production in the presence of sacrificial agents.

Figure 3b compares the hydrogen generation efficiency of pure and heat‐treated STO samples. STO‐P exhibited superior hydrogen generation compared to the heat‐treated samples, with STO‐3 outperforming STO‐24. The results suggest that smaller particle size of STO‐P, which results in a higher BET surface area, likely contributes to its enhanced catalytic efficacy. The catalytic performance of STO‐3, followed by STO‐24, decreased as particle size increased, reducing surface area.

Figure 3c–e depicts the flexocatalytic degradation of RhB dye using STO‐P, STO‐3, and STO‐24, respectively, under similar ultrasonic vibrational conditions. The data shows that STO‐P achieves the highest dye degradation, followed by STO‐3 and STO‐24, as indicated by the relative absorbance of dye degradation over time (Figure 3f). STO‐P exhibited the highest dye degradation efficiency, with ≈94% degradation and a rate constant of k = 1.05 × 10^−2^ at a catalyst concentration of 0.5 mg mL^−1^. In contrast, STO‐3 and STO‐24 achieved significantly lower degradation rates, ≈53% and 18%, respectively. The reaction rates for dye degradation were calculated using Langmuir‐Hinshelwood (L‐H) equations, and the reaction rate constants (k) were obtained from a plotting of ln(C/C_0_) as a function of time, as shown in Figure 3g.^[^ ^47^ ^]^ The flexocatalytic degradation of organic dyes can be understood through a series of chemical reactions, as detailed in (Equations (8), (9), (10)). This process builds upon the phenomenon described in (Equation 1), where ultrasonication induces the formation of charge carriers (holes h+ and electrons e‐) on the surface of STO. These charge carriers then engage with surrounding water molecules and oxygen, triggering the production of reactive oxygen species (ROS), primarily 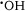 and superoxide anions (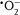).^[^ ^48^ ^]^ These highly reactive ROS possess the capability to rapidly attack and break down organic dye molecules, effectively degrading them into simpler compounds.

(8) $$e^{-}+O_{2}\to O_{2}^{•-}$$

(9) $$h^{+}+H_{2}O\to^{•}\mathrm{OH}$$

(10)

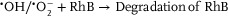




### 2.3 Stability of the Catalyst Structure

**Figure 4:**
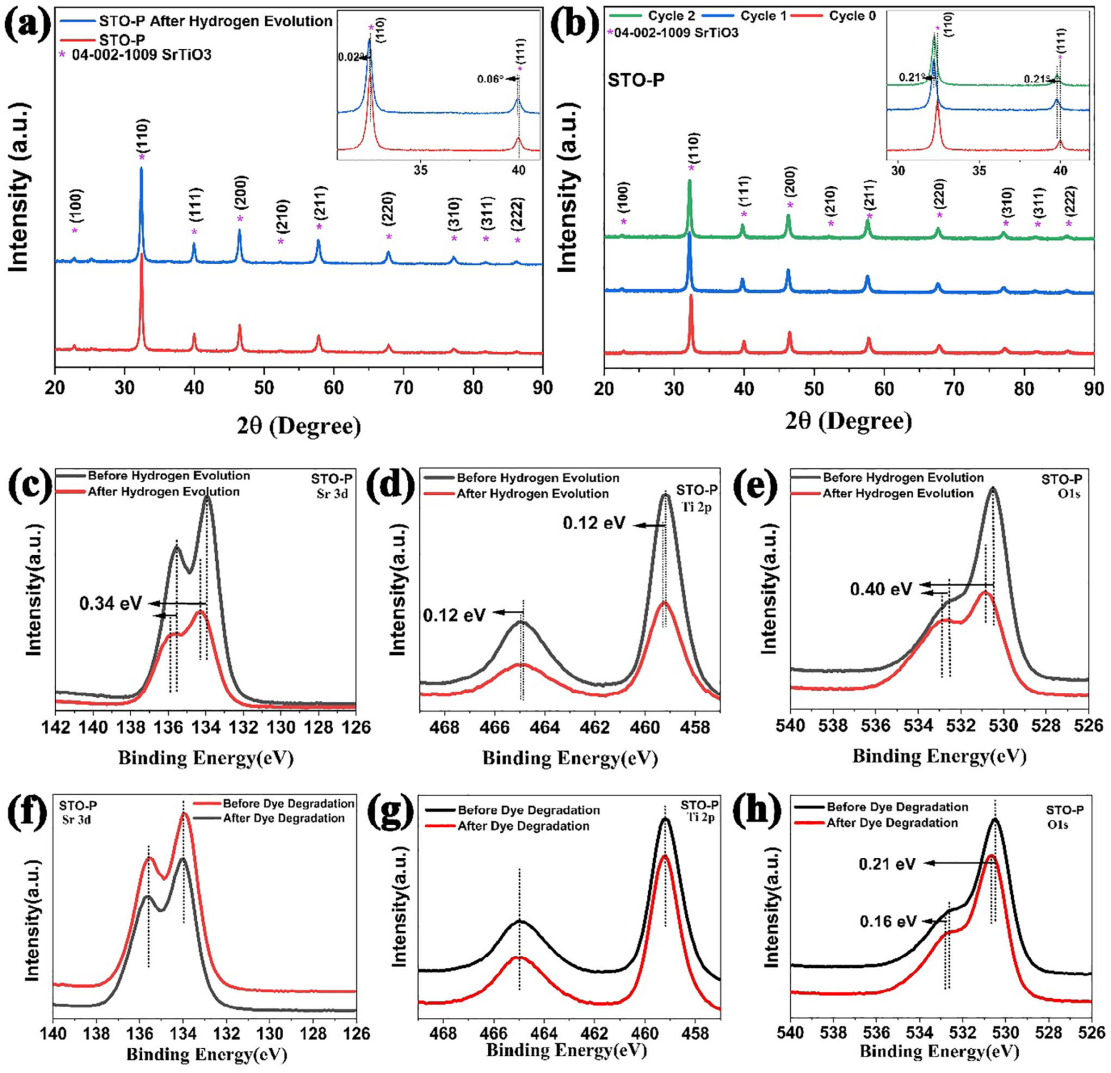
Structural and chemical analysis of STO before and after catalytic processes to evaluate material stability. a) XRD peaks of STO‐P before and after hydrogen generation of 4 h. b) XRD peaks of recycled STO‐P nano‐powders before and after 2 cycles of organic degradation c–e) XPS spectra of Sr 3d, Ti 2p, and O 1s states of STO‐P showing relative peaks position before and after hydrogen generation. f–h) XPS spectra of Sr 3d, Ti 2p, and O 1s states of STO‐P respectively showing relative peaks position before and after of organic dye degradation.

After the catalysis process, the stability of the STO‐P nano‐powder was evaluated. **Figure** **4**a,b shows the XRD patterns of pristine STO, both before and after hydrogen evolution and organic degradation. The XRD peaks of the recycled STO nanoparticles after the hydrogen evolution experiments (Figure 4a) display the same peak positions, with a negligible leftward shift (inset), and consistent intensities. This confirms the excellent structure stability of the catalyst after the hydrogen evolution processes.

In the dye degradation studies, a slight leftward shift (0.21°) was observed in the XRD peaks of the recycled STO‐P nanoparticles after the first cycles (Figure 4b) as shown in inset. However, no further shift was detected after the second cycle. This initial shift may be attributed to lattice relaxation caused by the functionalization of STO‐P with organic groups from the dye during the first cycle. The absence of additional shifts after the second cycle suggests that STO‐P may have been saturated with functional groups after the first cycle, stabilizing the lattice structure for subsequent cycles.

The XPS spectra of STO‐P, both pre and post‐hydrogen evolution (Figure 4c–e) and following dye degradation (Figure 4f–h), provide further insights into the stability of the STO‐P nano‐powders. As shown in Figure 4c–e, the Sr 3d, Ti 2P, and O1s states in STO‐P exhibit a noticeable shift toward higher binding energies after hydrogen evolution. This shift can be attributed to various factors associated with alterations in the surface chemistry and structure of STO. Potential causes include surface reduction, defect formation, and strain effects, all of which can influence the XPS spectra. Furthermore, the 4 h ultrasonic vibration treatment in DI water leads to the hydroxylation of STO‐P, modifying the chemical environment of the involved elements. These observations collectively underscore the complex surface dynamics of STO‐P nano‐powders during catalytic processes and highlight the importance of considering surface modifications in understanding their performance and stability.^[^ ^49^ ^]^

A detailed analysis of the O1s spectra, before and after hydrogen evolution and organic dye degradation (Figure S6a,b,c, Supporting Information), shows that the peak area corresponding to absorbed OH^−^ is significantly larger in the STO‐P after hydrogen evolution and dye degradation compared to the pristine STO. This suggests an increase in surface hydroxylation, which is attributed to extended exposure to ultrasonication. In contrast, the XPS spectra of STO‐P after dye degradation show only a minor shift toward higher energy in the O1s spectra (Figure 4h), while no shift is observed in the Sr 3d (Figure 4f) and Ti 2p (Figure 4g) spectra. This observation is further supported by the nearly identical deconvoluted spectras (Figure S7, Supporting Information).

Despite these surface modifications, XRD and XPS data indicate no significant structural changes in the recycled STO‐P. This is further confirmed by the hydrogen evolution and dye degradation experiments, which show that the catalytic performance of recycled STO‐P remains stable. The rate and quantity of hydrogen produced by both pure and recycled STO‐P are unchanged, as shown in Figure S4 (Supporting Information), which compares hydrogen production before and after the catalytic process. Similarly, the functionalization of STO‐P during the dye degradation has a minimal impact on the reaction kinetics and overall dye degradation efficiency, as depicted in Figure S5a,b (Supporting Information).

### 2.4 Mechanism Discussion

**Figure 5:**
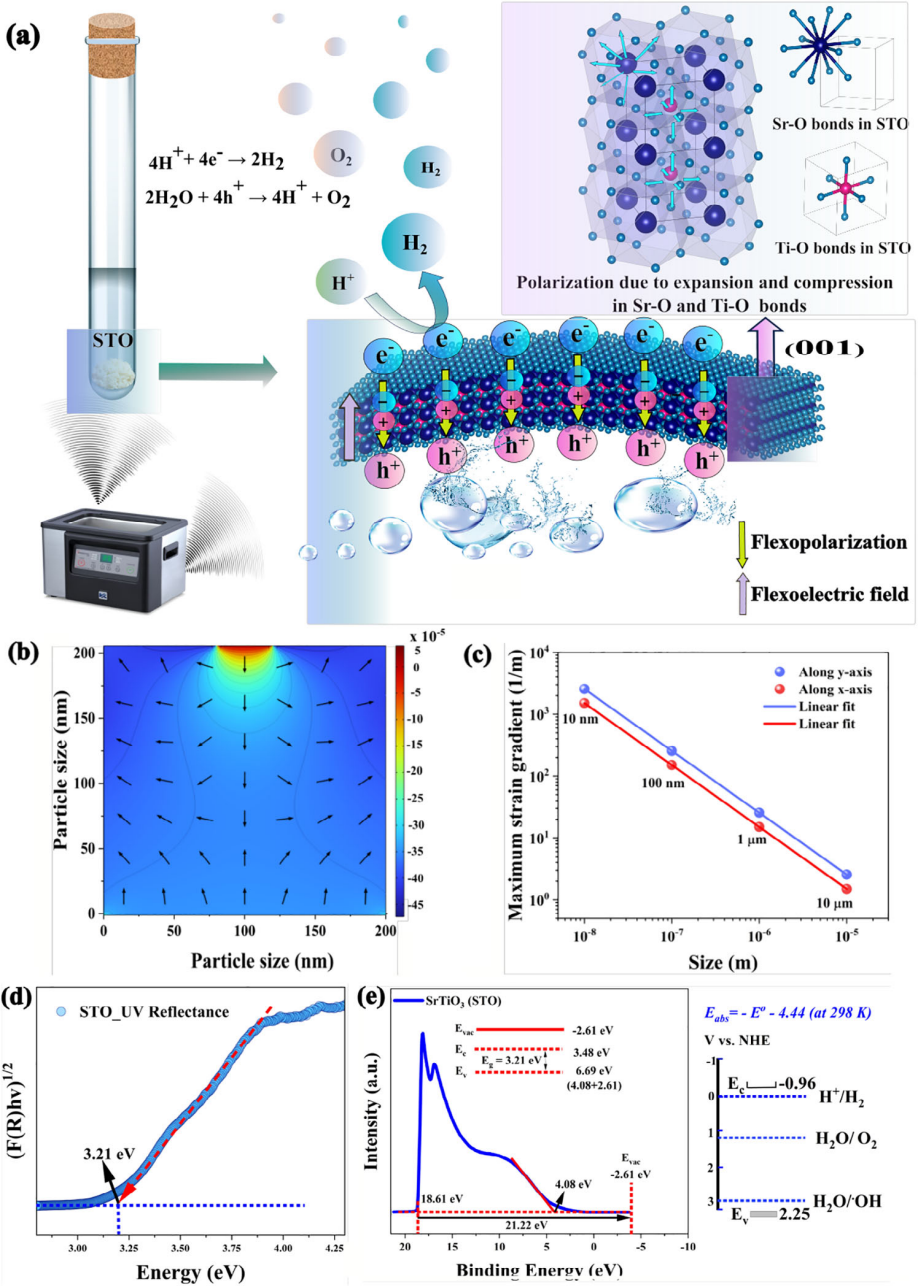
Mechanistic and theoretical insights into flexocatalysis and band structure properties of SrTiO_3_. a) Schematic illustration of the flexocatalytic mechanism of STO during hydrogen generation by ultrasonic vibration b) Theoretical simulations of flexoelectricity in STO using a tip‐force model, showing strain distribution and induced flexoelectric polarization at a stress value of 100 MPa. c) Maximum strain gradient changes with the size of the STO d) Kubelka‐Munk plot derived from DRS by UV–vis spectroscopy. e) Energy diagram of pure STO, based on the energy bandgap and UPS spectrum showing reduction potential of STO nanopowder.

Semiconductor nanoparticles with centrosymmetric structures, such as STO, typically lack piezoelectricity but often exhibit flexoelectric polarization. This polarization arises from the strain gradients due to non‐uniform stress distributions, known as flexoelectric effect.^[^ ^24^, ^50^ ^]^ In this study, the strain in STO nano‐powders was generated by ultrasonic bubble cavitation.^[^ ^48^ ^]^ **Figure** **5**a illustrates the flexocatalysis mechanism observed in the centrosymmetric STO nano‐powder.

When STO is subjected to the rupture of cavitation bubbles under ultrasonic vibrations, a strain field is induced throughout the crystal structure, leading to anisotropic expansion and compression of the Sr─O and Ti─O bonds. This mechanical deformation perturbs the local charge equilibrium, generating a transient flexoelectric polarization field. The periodic formation and collapse of cavitation bubbles create dynamic strain gradients that continuously stimulate charge redistribution within the STO lattice, reinforcing the internal electric field. In this process, electron‐hole pairs are generated through ultrasonic excitation and are separated by the transient flexoelectric field. Within the spatial continuum, these thermally excited electrons and holes are disentangled by the cross‐body flexoelectric polarization, subsequently migrating antipodally across the surface. This charge separation mechanism minimizes recombination losses, improving catalytic efficiency. This migration establishes a significant electrostatic potential gradient, effectively drawing ionic species from the surrounding medium and orchestrating intricate interfacial redox reactions. Protons, H⁺ undergo reduction to form molecular hydrogen, while oxygen molecules are transformed into 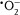, vital for the oxidative degradation of organic pollutants. This process not only enhances catalytic efficiency but also achieves refined localized charge redistribution and subtle electronic modulation, thereby optimizing the reaction dynamics at the interface for improved overall performance.^[^ ^48^ ^]^ During periodic ultrasonic irradiation, cavitation bubbles are engendered and undergo volumetric expansion in the rarefaction phase, followed by nonlinear oscillatory behavior and culminating in implosive collapse. This dynamic sequence precipitates a multitude of physical phenomena, including the generation of high‐velocity micro‐jets, the propagation of intense shock waves, and the exertion of substantial shear forces, collectively contributing to the continuous stimulation and modulation of catalytic surfaces.^[^ ^51^ ^]^

In this study, ultrasonic vibration (200 W, 40 kHz) produced cavitation bubbles, applying mechanical force to the STO nano‐powder. The pressure generated ranged from 10^5^ to 10^6^ Pa in the surrounding liquid medium,^[^ ^52^ ^]^ reaching 10^8^ to 1,0^9^ Pa when cavitation bubbles ruptured at a critical size.^[^ ^53^, ^54^ ^]^ This cavitation‐induced flexoelectric polarization mimics the piezocatalysis effect seen in non‐centrosymmetric materials,^[^ ^27^ ^]^ enhancing electron mobility and contributing to STO's catalytic performance.

The size‐dependent nature of flexocatalysis, where smaller particle sizes improve catalytic activity, was confirmed through hydrogen evolution experiments (Figure 3b). Reducing the average STO size from 117 nm to 35 nm resulted in a 3.32‐fold increase in hydrogen production. Similarly, the dye degradation rate improved significantly from 18% after 180 min to 80% after just 60 min of ultrasonic treatment (Figure 3f). A 2D tip force model was used to explore the strain gradient and polarization in STO nanoparticles, applying a 100 MPa force to a 200 nm wide STO particle with a fixed bottom boundary. The flexoelectric polarization and strain gradient distributions (Figure 5b) were calculated using published flexoelectric tensor values for STO‐P (µ₃₃₃₃ = 0.2 nC m^−1^, µ₃₃₁₁ = 0.2 nC m^−1^, µ₁₃₁₃ = 0.2 nC m^−1^).^[^ ^7^, ^55^ ^]^ The highest polarization occurred in the transverse plane (48.02 nC m^−^^2^), followed by the longitudinal (39.02 nC m^−^^2^) and shear planes (1.37 nC m^−^^2^). The effect of particle size on strain gradient was modeled by varying particle dimensions from 10 nm to 10 µm. The relationship followed the equation S' = kL⁻¹, where S' represents the strain gradient, L is particle size, and k is a scaling factor, confirming the inverse proportionality between particle size and strain gradient (Figure 5c).

Finally, the energy bandgap of STO was determined using diffuse reflectance spectroscopy (DRS) and the Kubelka‐Munk function, yielding a value of 3.21 eV (Figure 5d). The conduction band minimum of STO (−0.96 V vs NHE) is more negative than the H^+^/H_2_ redox potential (0.00 V), confirming STO's capability to reduce H^+^ to H_2_ in this experimental setup (Figure 5e).^[^ ^56^ ^]^

Though flexoelectricity is the primary mechanism in our system, it is worth considering the potential contribution of tribocatalysis. Tribocatalysis arises from the friction between materials and can generate active sites on the catalyst surface. In our experiments, the friction between STO nanoparticles during ultrasonication could potentially induce tribocatalysis, contributing to the observed catalytic activity. However, several factors suggest that tribocatalysis plays a limited role in our system. First, the dynamic fluid environment and cavitation‐induced shockwaves limit sustained solid‐solid contact, which is essential for effective tribocatalysis.^[^ ^51^ ^]^ Second, our results show a strong size‐dependent enhancement of catalytic activity, which is a characteristic feature of flexoelectricity, not tribocatalysis.^[^ ^16^ ^]^ If tribocatalysis were a major contributor, we would expect factors like particle surface properties and contact frequency to play a more significant role than size.^[^ ^57^ ^]^ Finally, the limited opportunity for triboelectric charge generation in our system further supports the notion that tribocatalysis is not the dominant mechanism. Therefore, we believe that the contribution of tribocatalysis, if any, is likely to be minor compared to the dominant flexoelectric effect. Further investigation is needed to fully decouple the effects of these two mechanisms and to determine their relative contributions to the overall catalytic activity.

While flexocatalysis with sacrificial agents and piezocatalysis in pure water have been widely studied, flexocatalytic water splitting using pure water remains largely unexplored. **Table** **1** highlights recent advances in mechano‐catalytic water splitting with various catalysts, without the use of sacrificial agents, and their hydrogen production efficiency. Notably, our research shows that centrosymmetric STO exhibits exceptional flexocatalytic hydrogen evolution in pure water. This discovery represents a significant step forward in mechano‐catalytic water splitting, paving the way for more sustainable and efficient hydrogen production technologies.

**Table 1:** Pure water splitting by different catalysts using ultrasonic vibrations.

Catalysts	Particle size	Ultrasonic Vibration	Catalytic activity (P: piezocatalysis; F: flexocatalysis)	Hydrogen Evolution [µmolg^−1^ h^−1^]	Refs.
SrTiO_3_ Nanoparticles	35 nm	40 kHz, 200 W	F	1289.53	This work
Rutile TiO_2_	50 nm	40 KHz, 200 W	F	2380	[16]
Barium dititanate (BaTi_2_O_5_) Nanocrystals	10‐20 nm	40 KHz, 200 W	F	1160	[58]
2D Carbon Nitride	4.0 nm (thickness)	700 W	P	1190	[59]
Bi2Fe_4_O_9_ Nanoplates	150–180 nm (Thickness) 500–800 nm (Width)	40 kHz, 200 W	P	1058	[60]
Co_4_N–WNx composite	–	40 kHz, 120 W	P	262.7	[61]
BiFeO_3_ Nanosheets	380 nm	100 W	P	124.1	[62]
BaTiO_3_ Nanosheets	200 nm	40 kHz, 100 W	P	92	[63]
Defect‐rich MoS_2_	–	40 kHz, 150 W	P	47.75	[64]
SrSn_0.05_Ti_0.95_O_3_	–	40 kHz, 80 W	P	101.46	[65]
Ultra‐thin ZnS NS	50 nm	40 kHz	P	1.08	[66]
BaTiO_3_ Nanoparticles	–	40 kHz	P	159	[67]
N‐TiO_2_@C NSs	2.0 nm (Thickness)	40 kHz, 200 W	P	390	[68]
Defective BaTiO_3–x_ Nanoparticles	30‐60 nm	40 kHz, 100 W	P	96.9	[69]
BaTiO_3_ Nanoparticles	10 nm	60 kHz	P	655	[70]
Bi_1/2_Na_1/2_TiO_3_	–	40 kHz, 100 W	P	506.7	[71]
Bi_0.5_(Na_1−x_K_x_)_0.5_TiO_3_ Nanoparticles	–	40 kHz, 400 W	P	77.0	[72]

## 3 Conclusion

In conclusion, we have demonstrated a flexocatalytic effect in centrosymmetric STO nano‐powder driven by ultrasonic vibration and bubble cavitation, attributed to variations in flexoelectric polarization within the material. Among the samples tested, pristine STO exhibited the highest catalytic efficiency, achieving hydrogen evolution rates of 1289.53 µmol g^−1^ h^−1^ in pure water after 4 h of ultrasonic vibration, and organic degradation of ≈94% within 3 h under the same conditions in the dark. The superior performance of the pristine STO is linked to its smaller particle size compared to larger particle size of heat‐treated samples, which enhances catalytic activity. The high efficiency, strong flexocatalytic behavior, reusability, and durability of STO‐P underscore its potential in hydrogen production and organic pollutant degradation. These findings open new possibilities for advancing flexocatalysis and improving catalytic systems in future research and technological applications.

## 4 Experimental Section

### Materials

SrTiO3 nano‐powder (99.5%) was obtained from Shanghai Macklin Biochemical Co. Ltd., Shanghai, China. The nano‐powder was subjected to heat treatment at 1000 °C for 3 and 24 h in air to study the effect of particle size on its flexocatalytic activities.

### Material Characterization

XRD was used to identify the crystal structure and phase of the commercial and the heat‐treated materials. XRD patterns were obtained using a PANalytical Aeris XRD system using Cu Kα radiation (*λ* = 0.15418 nm). Rietveld refinement of the XRD patterns was performed using GSAS II software, and the structural models of the refined phases were visualized using the 3D visualization program VESTA 3.

The morphologies of the materials were analyzed using TEM. Specimens were prepared by drop‐casting onto carbon‐coated copper grids, and images were acquired using a JEM‐F200 field emission transmission electron microscope (JEOL). Particles size distribution was measured from TEM images using ImageJ software. HRTEM images and SAED patterns were used to measure the d‐spacing value of the samples with the Gatan Microscopy Suite (GMS 3) software.

Surface area analysis was performed using a TriStar II surface area and porosity analyzer (Micromeritics Instrument Corp., Norcross, GA, USA). This analyser measures multipoint BET surface area by nitrogen gas as adsorption‐desorption at 77K.

Raman spectroscopy was conducted to further investigate phase changes following heat treatment. Raman spectra were obtained using a LabRAM HR Evolution system (Horiba) with a 633 nm laser, and a 300 grooves per mm grating.

A Nexsa X‐ray photoelectron spectrometer and ultraviolet photoelectron spectrometer (UPS) from Thermo Fisher Scientific were utilized to analyse the surface chemical states and work function of the sample powders. The UPS results were calibrated using silver, Ag with a work function of 4.27 eV, and a negative bias voltage of 10 V was applied to the sample holder for improved signal strength.

DRS of the sample powders were measured using ultraviolet‐visible (UV‐Vis) spectroscopy (Shimadzu UV‐3600) with an integrated sphere, scanning from 200 to 600 nm to evaluate the energy bandgap (*E*g).

### COMSOL Simulation

COMSOL Multiphysics simulations were used to model the tip force in 2D to understand the flexoelectric behavior of STO. This static model simulates the volumetric strain distribution and the strain‐gradient induced flexoelectric polarization under a fixed constraint. A tip force equivalent to 100 MPa was applied in the center of the top boundary of a 2D STO square with a width of 200 nm. The Poisson's ratio was set to 0.25, and Young's modulus was set to 300 GPa for the solid STO. The bottom boundary was fixed, and only the elastic strain was considered in the simulation. Flexoelectric polarization (***P**_l_*) was calculated by considering the volumetric strain gradient (ε_*v*_) along the coordinate axis and the corresponding flexoelectric coefficient (µ_*i*_), as shown in (Equation 11).

(11) $$P_{l}=\mu_{i}\nabla \varepsilon_{v}$$

### Hydrogen Evolution Experiment

Hydrogen evolution was analyzed offline, with gas samples injected using a 1 mL Hamilton microsyringe (1000 series GASTIGHT, Sigma‐Aldrich) into a gas chromatograph (GC 2600, China) equipped with a thermal conductivity detector for enhanced analytical precision. The GC column temperature was maintained at 150 °C and 120 °C with argon as the carrier gas. The retention time for hydrogen detection was 5.5 min, ensuring accurate quantification.

The hydrogen generation experiment was conducted under two conditions: with and without a sacrificial agent. A 0.05 M aqueous solution of Na_2_SO_3_ was used as the sacrificial agent. For catalytic hydrogen evolution of hydrogen, 2 mg of SrTiO_3_ nanoparticles were dispersed in 10 mL of either DI water or an aqueous solution containing the sacrificial agent in a sealed 45 mL borosilicate glass tube. The tube was thoroughly purged with high‐purity Ar (≥ 99.99%) for 10 min using a gas dispersion tube to remove any residual oxygen, ensuring an oxygen‐free inert atmosphere. After purging, the tube was immediately sealed with a gas‐tight rubber septum and secured with an aluminium crimp cap to prevent external contamination.

The sealed tube was placed in an ultrasonic bath (40KHz, 200 W), which induced mechanical stress on the nanoparticle surface through the acoustic power generated by the collapse of cavitation bubbles. The water level in the ultrasonic bath was maintained at specific height, ensuring uniform energy distribution. A light‐shielding cover was used to eliminate any photochemical effects, ensuring that hydrogen evolution was purely mechanochemical. Ice bags were placed around the bath, and a thermocouple probe monitored and maintained the temperature at 25 °C. Gas samples (1 mL each) were collected every hour using a Hamilton microsyringe through the septum. Three independent trials were conducted for each condition to ensure reproducibility. The collected gas samples were analyzed via GC, and hydrogen was quantified using a pre‐calibrated standard curve.

### Organic Dye Degradation

The flexocatalytic degradation of organic pollutants was carried out in the same ultrasonic bath used for the hydrogen evolution experiment, ensuring consistent cavitation conditions. In a standard procedure, 25 mg of catalysts (at a concentration of 0.5 g L^−1^) was dispersed in 50 mL of a 5 mg L^−1^ RhB solution, stirred for 1 h, and allowed to reach adsorption‐desorption equilibrium overnight. The prepared suspension was divided into five separate borosilicate glass tubes (10 mL each), sealed, and placed in the ultrasonic bath at a uniform height to ensure consistent exposure to acoustic energy. Samples (3 mL each) were collected every 30 min using a syringe. Temperature was controlled using ice bags, and a thermocouple monitored temperature fluctuations. Collected samples were centrifuged at 10000 RPM and 10 min to separate the catalyst from the solution. The supernatant was analyzed using UV–vis spectroscopy (Shimadzu UV‐1900), and the absorbance of RhB was recorded at 554 nm. Control experiments were performed with RhB solution without catalyst to assess non‐catalytic degradation. Three independent trials were conducted to validate reproducibility.

## Conflict of Interest

The authors declare no conflict of interest.

## Supporting information



Supporting Information

## Data Availability

The data that support the findings of this study are available from the corresponding author upon reasonable request.

## References

[1] M. Yue , H. Lambert , E. Pahon , R. Roche , S. Jemei , D. Hissel , Renew. Sustain. Energy Rev. 2021, 146, 111180.

[2] R. Hren , A. Vujanović , Y. Van Fan , J. J. Klemeš , D. Krajnc , L. Čuček , Renew. Sustain. Energy Rev. 2023, 173, 113113.

[3] Y. Sun , X. Li , A. Vijayakumar , H. Liu , C. Wang , S. Zhang , Z. Fu , Y. Lu , Z. Cheng , ACS Appl. Mater. Interfaces 2021, 13, 11050. 33634697 10.1021/acsami.1c01407

[4] N. Meng , W. Liu , R. Jiang , Y. Zhang , S. Dunn , J. Wu , H. Yan , Prog. Mater. Sci. 2023, 138, 101161.

[5] Y. Meng , G. Chen , M. Huang , Nanomaterials 2022, 12, 1171. 35407289 10.3390/nano12071171 PMC9000841

[6] Z. L. Wang , Nanopiezotron. Adv. Mater. 2007, 19, 889.

[7] L. Wang , S. Liu , X. Feng , C. Zhang , L. Zhu , J. Zhai , Y. Qin , Z. L. Wang , Nat. Nanotechnol. 2020, 15, 661. 32572230 10.1038/s41565-020-0700-y

[8] S. Krichen , P. Sharma , J. Appl. Mech. 2016, 83, 030801.

[9] L. Shu , R. Liang , Z. Rao , L. Fei , S. Ke , Y. Wang , J. Adv. Ceram. 2019, 8, 153.

[10] D. Lee , A. Yoon , S. Y. Jang , J.‐G. Yoon , J.‐S. Chung , M. Kim , J. F. Scott , T. W. Noh , Phys. Rev. Lett. 2011, 107, 057602. 21867099 10.1103/PhysRevLett.107.057602

[11] P. Zubko , G. Catalan , A. Buckley , P. R. L. Welche , J. F. Scott , Phys. Rev. Lett. 2007, 99, 167601. 17995293 10.1103/PhysRevLett.99.167601

[12] D. Lee , T. W. Noh , Philosophic. Trans. Royal Soc. A, Mathemat., Phys. Eng. Sci. 2012, 370, 4944. 10.1098/rsta.2012.0200 22987037

[13] Y. Lun , X. Wang , J. Kang , Q. Ren , T. Wang , W. Han , Z. Gao , H. Huang , Y. Chen , L.‐Q. Chen , D. Fang , J. Hong , Adv. Mater. 2023, 35, 2302320. 10.1002/adma.202302320 37358059

[14] G. Dong , S. Li , M. Yao , Z. Zhou , Y.‐Q. Zhang , X. Han , Z. Luo , J. Yao , B. Peng , Z. Hu , H. Huang , T. Jia , J. Li , W. Ren , Z.‐G. Ye , X. Ding , J. Sun , C.‐W. Nan , L.‐Q. Chen , J. Li , M. Liu , Science 2019, 366, 475. 31649196 10.1126/science.aay7221

[15] T. Wu , K. Liu , S. Liu , X. Feng , X. Wang , L. Wang , Y. Qin , Z. L. Wang , Adv. Mater. 2023, 35, 2208121. 10.1002/adma.202208121 36333880

[16] Y. Du , S. Zhang , Z. Cheng , Nano Energy 2024, 127, 109731.

[17] M. O. Olagunju , X. Poole , P. Blackwelder , M. P. Thomas , B. S. Guiton , D. Shukla , J. L. Cohn , B. Surnar , S. Dhar , E. M. Zahran , L. G. Bachas , M. R. Knecht , ACS Appl. Nano Mater. 2020, 3, 4904.

[18] L. F. da Silva , O. F. Lopes , V. R. de Mendonça , K. T. G. Carvalho , E. Longo , C. Ribeiro , V. R. Mastelaro , Photochem. Photobiol. 2016, 92, 371. 27010848 10.1111/php.12586

[19] A. Vijay , S. Vaidya , ACS Appl. Nano Mater. 2021, 4, 3406.

[20] T. Fix , Y. Zakaria , D. Stoeffler , D. Muller , A. Dinia , A. Slaoui , Sol. RRL 2024, 8, 2400237.

[21] M. Asif , A. Afaq , M. Amin , K. Raouf , A. Majeed , M. Asif , Mater. Today Commun. 2023, 37, 106966.

[22] Z. Liu , W. Lu , S. Zeng , J. Deng , Z. Huang , C. Li , C. J. Li , M. Motapothula , W. M. Lü , L. Sun , K. Han , J. Q. Zhong , P. Yang , N. N. Bao , W. Chen , J. S. Chen , Y. P. Feng , J. M. D. Coey , T. Venkatesan , Ariando , arXiv 2014, 14046863.

[23] H. J. Clabel , J. Chacaliaza‐Ricaldi , E. Marega Jr. , Front. Nanotechnol. 2022, 4, 827925.

[24] U. K. Bhaskar , N. Banerjee , A. Abdollahi , Z. Wang , D. G. Schlom , G. Rijnders , G. Catalan , Nat. Nanotechnol. 2016, 11, 263. 26571008 10.1038/nnano.2015.260

[25] G. Kolhatkar , M. Nicklaus , A. Hadj Youssef , C. Cojocaru , M. Rivard , A. Merlen , F. Légaré , A. Ruediger , Adv. Funct. Mater. 2019, 29, 1901266.

[26] D. E. Grupp , A. M. Goldman , Science 1997, 276, 392. 9103192 10.1126/science.276.5311.392

[27] J. Ling , K. Wang , Z. Wang , H. Huang , G. Zhang , Ultrason. Sonochem. 2020, 61, 104819. 31669844 10.1016/j.ultsonch.2019.104819

[28] Y. Jiang , J. Xie , Z. Lu , J. Hu , A. Hao , Y. Cao , J. Colloid Interface Sci. 2022, 612, 111. 34983011 10.1016/j.jcis.2021.10.170

[29] M. I. Ahmad , S. S. Bhattacharya , Appl. Phys. Lett. 2009, 95, 191906.

[30] S. Komai , M. Hirano , N. Ohtsu , Surf. Interface Anal. 2020, 52, 823.

[31] M. Shang , H. Hu , G. Lu , Y. Bi , J. Mater. Chem. A 2016, 4, 5849.

[32] K. Aravinthkumar , E. Praveen , J. R. Mary , C. R. Mohan , Inorg. Chem. Commun. 2022, 140, 109451.

[33] H. Idriss , Surf. Sci. 2021, 712, 121894.

[34] W. G. Nilsen , J. G. Skinner , J. Chem. Phys. 1968, 48, 2240.

[35] Y. I. Yuzyuk , Phys. Solid State 2012, 54, 1026.

[36] R. Sasikala , O. D. Jayakumar , S. K. Kulshreshtha , Ultrason. Sonochem. 2007, 14, 153. 16904930 10.1016/j.ultsonch.2006.06.005

[37] Y. Iida , K. Yasui , T. Tuziuti , M. Sivakumar , Microchem. J. 2005, 80, 159.

[38] Y. Wang , Y. Xu , S. Dong , P. Wang , W. Chen , Z. Lu , D. Ye , B. Pan , D. Wu , C. D. Vecitis , G. Gao , Nat. Commun. 2021, 12, 3508. 34108484 10.1038/s41467-021-23921-3 PMC8190189

[39] P. Khare , R. K. Patel , S. Sharan , R. Shankar , M. P. Shah , Advanced Oxidation Processes for Effluent Treatment Plants, Elsevier, Amsterdam 2021. p. 137.

[40] K.‐S. Hong , H. Xu , H. Konishi , X. Li , J. Phys. Chem. C 2012, 116, 13045.

[41] S. Li , X. Zhang , F. Yang , J. Zhang , W. Shi , F. Rosei , Chem. Catal. 2024, 4, 100901.

[42] J. Cho , M. Kim , K. T. Park , C. H. Rhee , H. W. Park , B. Koo , J. C. Jung , Mol. Catal. 2023, 550, 113536.

[43] Z. Bao , V. Fung , F. Polo‐Garzon , Z. D. Hood , S. Cao , M. Chi , L. Bai , D. E. Jiang , Z. Wu , J. Catal. 2020, 384, 49.

[44] P. Yu , K. Zhang , H. Huang , M. Wen , Q. Li , W. Zhang , C. Hu , W. Zheng , Appl. Surf. Sci. 2017, 410, 470.

[45] R. Chandra Das , B. M. L. Chaki , R. Sluyter , M. Lerch , K. Konstantinov , Mater. Lett. 2023, 346, 134523.

[46] C. Huang , C. A. Linkous , O. Adebiyi , A. T‐Raissi , Environ. Sci. Technol. 2010, 44, 5283. 20515046 10.1021/es903766w

[47] D. Ayodhya , M. Venkatesham , A. Santoshi kumari , G. B. Reddy , D. Ramakrishna , G. Veerabhadram , J. Exp. Nanosci. 2016, 11, 418. 10.1007/s10895-015-1639-5 26275559

[48] Z. Liu , X. Wen , Y. Wang , Y. Jia , F. Wang , G. Yuan , Y. Wang , Adv. Mater. Technol. 2022, 7, 2101484.

[49] J.‐H. Jhang , J. A. Boscoboinik , E. I. Altman , J. Chem. Phys. 2020, 152, 084705. 32113358 10.1063/1.5142621

[50] S. Das , B. Wang , T. R. Paudel , S. M. Park , E. Y. Tsymbal , L.‐Q. Chen , D. Lee , T. W. Noh , Nat. Commun. 2019, 10, 537. 30710079 10.1038/s41467-019-08462-0 PMC6358620

[51] M. Ashokkumar , Ultrason. Sonochem. 2011, 18, 864. 21172736 10.1016/j.ultsonch.2010.11.016

[52] H. Li , Y. Sang , S. Chang , X. Huang , Y. Zhang , R. Yang , H. Jiang , H. Liu , Z. L. Wang , Nano Lett. 2015, 15, 2372. 25803813 10.1021/nl504630j

[53] E. B. Flint , K. S. Suslick , Science 1991, 253, 1397. 17793480 10.1126/science.253.5026.1397

[54] D. J. Flannigan , K. S. Suslick , Nature 2005, 434, 52. 15744295 10.1038/nature03361

[55] T. Xu , J. Wang , T. Shimada , T. Kitamura , J. Phys., Condens. Matter 2013, 25, 415901. 24061150 10.1088/0953-8984/25/41/415901

[56] K. Xu , M. Yao , J. Chen , P. Zou , Y. Peng , F. Li , X. Yao , J. Alloys Compd. 2015, 653, 7.

[57] T. A. Burgo , C. A. Silva , L. B. Balestrin , F. Galembeck , Sci. Rep. 2013, 3, 2384. 23934227 10.1038/srep02384 PMC3740278

[58] Y. Du , W. Sun , X. Li , C. Hao , J. Wang , Y. Fan , J. Joseph , C. Yang , Q. Gu , Y. Liu , S. Zhang , Z. Cheng , Adv. Sci. 2024, 11, 2404483. 10.1002/advs.202404483 PMC11481254 39119840

[59] H. Tan , W. Si , W. Peng , X. Chen , X. Liu , Y. You , L. Wang , F. Hou , J. i Liang , Nano Lett. 2023, 23, 10571. 37929933 10.1021/acs.nanolett.3c03466

[60] Y. Du , T. Lu , X. Li , Y. Liu , W. Sun , S. Zhang , Z. Cheng , Nano Energy 2022, 104, 107919.

[61] J. Yu , H. Guo , W. Feng , X. Guo , Y. Zhu , T. Thomas , C. Jiang , S. Liu , M. Yang , Dalton Trans. 2022, 51, 7127. 35466976 10.1039/d2dt00381c

[62] H. You , Z. Wu , L. Zhang , Y. Ying , Y. Liu , L. Fei , X. Chen , Y. Jia , Y. Wang , F. Wang , S. Ju , J. Qiao , C.‐H. Lam , H. Huang , Angew. Chem. Int. Ed. Engl. 2019, 58, 11779. 31225687 10.1002/anie.201906181

[63] C. Yu , M. Tan , Y. Li , C. Liu , R. Yin , H. Meng , Y. Su , L. Qiao , Y. Bai , J. Colloid Interface Sci. 2021, 596, 288. 33839354 10.1016/j.jcis.2021.03.040

[64] R. Lei , F. Gao , J. Yuan , C. Jiang , X. Fu , W. Feng , P. Liu , Appl. Surf. Sci. 2022, 576, 151851.

[65] Z. Chen , W. Liu , L. Zheng , Q. Chen , Y. Liu , S. Lan , M. Zhu , Sep. Purif. Technol. 2025, 353, 128307.

[66] W. Feng , J. Yuan , L. Zhang , W. Hu , Z. Wu , X. Wang , X. Huang , P. Liu , S. Zhang , Appl. Catal., B 2020, 277, 119250.

[67] R. Su , Z. Wang , L. Zhu , Y. Pan , D. Zhang , H. Wen , Z.‐D. Luo , L. Li , F. T. Li , M. Wu , L. He , P. Sharma , J. Seidel , Angew. Chem., Int. Ed. 2021, 60, 16019. 10.1002/anie.202103112 33871146

[68] X. Zhao , X. Lu , W.‐J. Chen , M.‐Q. Yang , X. Pan , Z. Bian , J. Colloid Interface Sci. 2024, 659, 11. 38157722 10.1016/j.jcis.2023.12.101

[69] Y. Jiang , C. Y. Toe , S. S. Mofarah , C. Cazorla , S. L. Y. Chang , Y. Yin , Q. Zhang , S. Lim , Y. Yao , R. Tian , Y. Wang , T. Zaman , H. Arandiyan , G. G. Andersson , J. Scott , P. Koshy , D. WangCharles , C. Sorrell , ACS Sustain. Chem. Eng. 2023, 11, 3370.

[70] R. Su , H. A. Hsain , M. Wu , D. Zhang , X. Hu , Z. Wang , X. Wang , F.‐T. Li , X. Chen , L. Zhu , Y. Yang , Y. Yang , X. Lou , S. J. Pennycook , Angew. Chem., Int. Ed. 2019, 58, 15076. 10.1002/anie.201907695 31404487

[71] A. Ranjan , K.‐Y. Hsiao , C.‐Y. Lin , Y.‐H. Tseng , M.‐Y. Lu , ACS Appl. Mater. Interfaces 2022, 14, 35635. 35905439 10.1021/acsami.2c07817

[72] J. Liang , Y. Jiang , Y. Sun , A. Rawal , Q. Zhang , Z. Song , Y. Sakamoto , J. Du , C. Jiang , S. L. Y. Chang , L. Fei , S. Ke , Z. Chen , W. Li , D. Wang , J. Mater. Chem. A 2023, 11, 16093.

