# Supplementary material for: Flexocatalytic Hydrogen Generation and Organics Degradation by Nano SrTiO3
Source: Adv Sci (Weinh). 2025 Apr 25;12(23):2500034. doi: 10.1002/advs.202500034 (PMC12199342; doi:10.1002/advs.202500034)
Supplement: Supplementary file 1 — Supporting Information [file ADVS-12-2500034-s001.docx]

**Supporting Information**

**Flexocatalytic Hydrogen Generation and Organics Degradation by nano STO**

*Susmita Mondal, Rajib Chandra Das, Yumeng Du, Zhenyuan Hou, Konstantin Konstantinov, Zhenxiang Cheng**

Institute for Superconducting and Electronic Materials, Faculty of Engineering and Information Sciences, University of Wollongong, Squires Way, North Wollongong, NSW 2500, Australia

***Corresponding author**


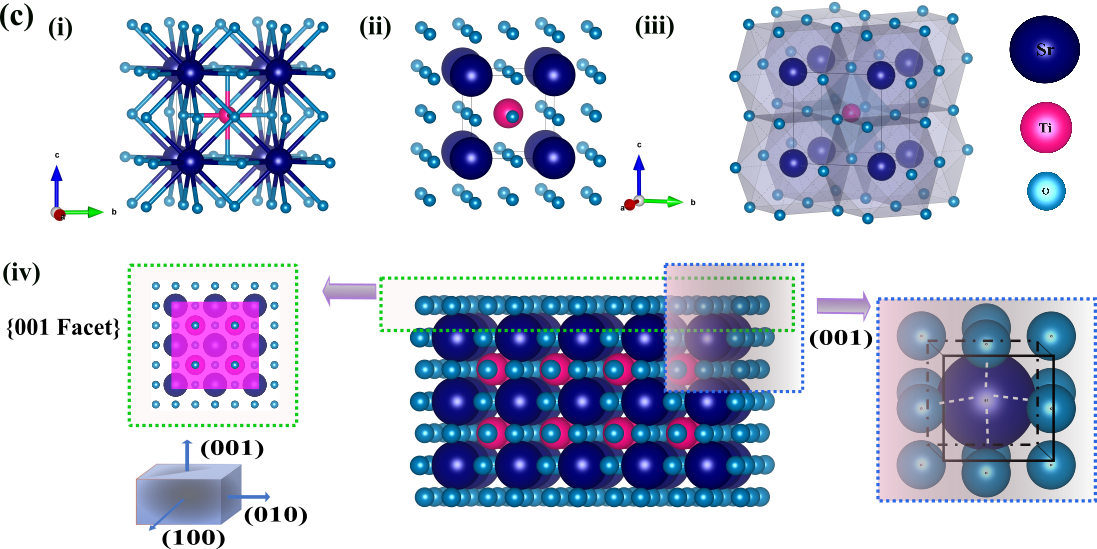


**Figure S1:** **(i-iii)** Centrosymmetric structure of STO and **(iv)** Lattice distribution of pure STO nanopowder along the (001) face.

Figure S1(i-iii) illustrates the centrosymmetric structure of STO, visualized using the 3D program VESTA 3 following Rietveld refinement. Figure S1(iv) shows the lattice distribution along the (001) face. In the STO unit cell, strontium (Sr) atoms occupy the corners, while titanium (Ti) atoms are centrally positioned. Sr^2+^ ions form SrO_12_ cuboctahedra by bonding with 12 equivalent O^2^- ions, and Ti^4+^ ions connect to six O^2-^ ions, creating TiO_6_ octahedra. These SrO_12_ cuboctahedra share 12 corners with other SrO_12_ units and 14 faces with 6 SrO_12_ cuboctahedra and 8 TiO_6_ octahedra. TiO_6_ octahedra shares 6 corners and 8 faces with SrO_12_ cuboctahedra. This symmetrical arrangement of positive and negative charges results in a net charge of zero, highlighting the balanced and stable structure of SrTiO_3_.

| 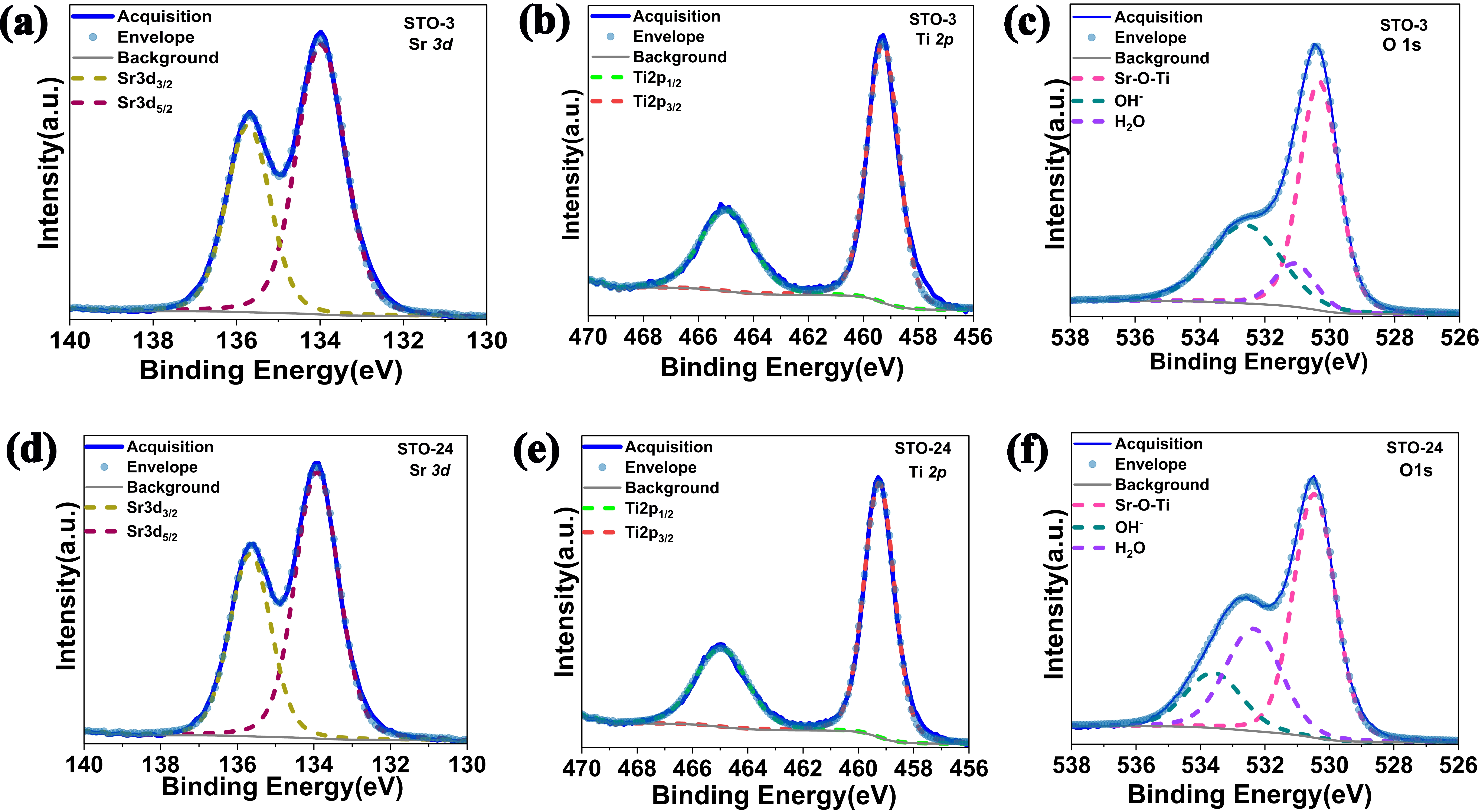 |
| --- |

**Figure S2: (a-c)** Deconvoluted XPS spectra of STO-3 presenting the individual components of the O 1s, Sr 3d, and Ti 2p peaks. **(d-f)** Deconvoluted XPS spectra of STO-24 presenting the individual components of the O 1s, Sr 3d, and Ti 2p peaks.

| **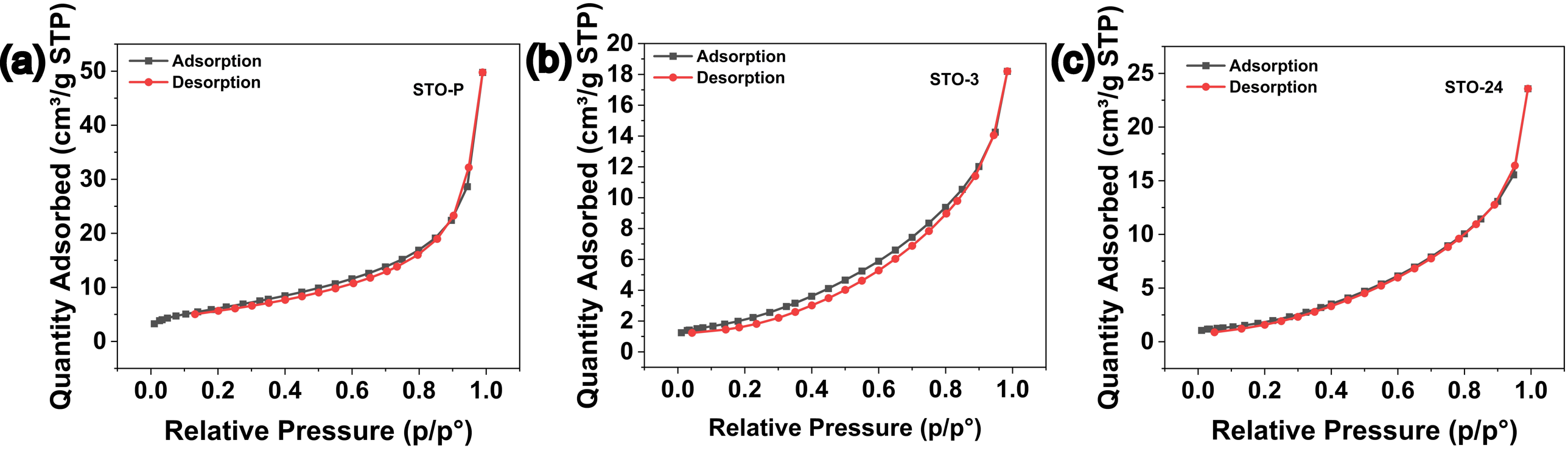** |
| --- |

**Figure S3:** Surface Area Evolution: BET Isotherms of STO-P **(a)**, STO-3 **(b)**, and STO-24 **(c)**.





**Figure** **S4:** Comparison of hydrogen production by the STO-P and recycled STO-P after the catalytic process.

| 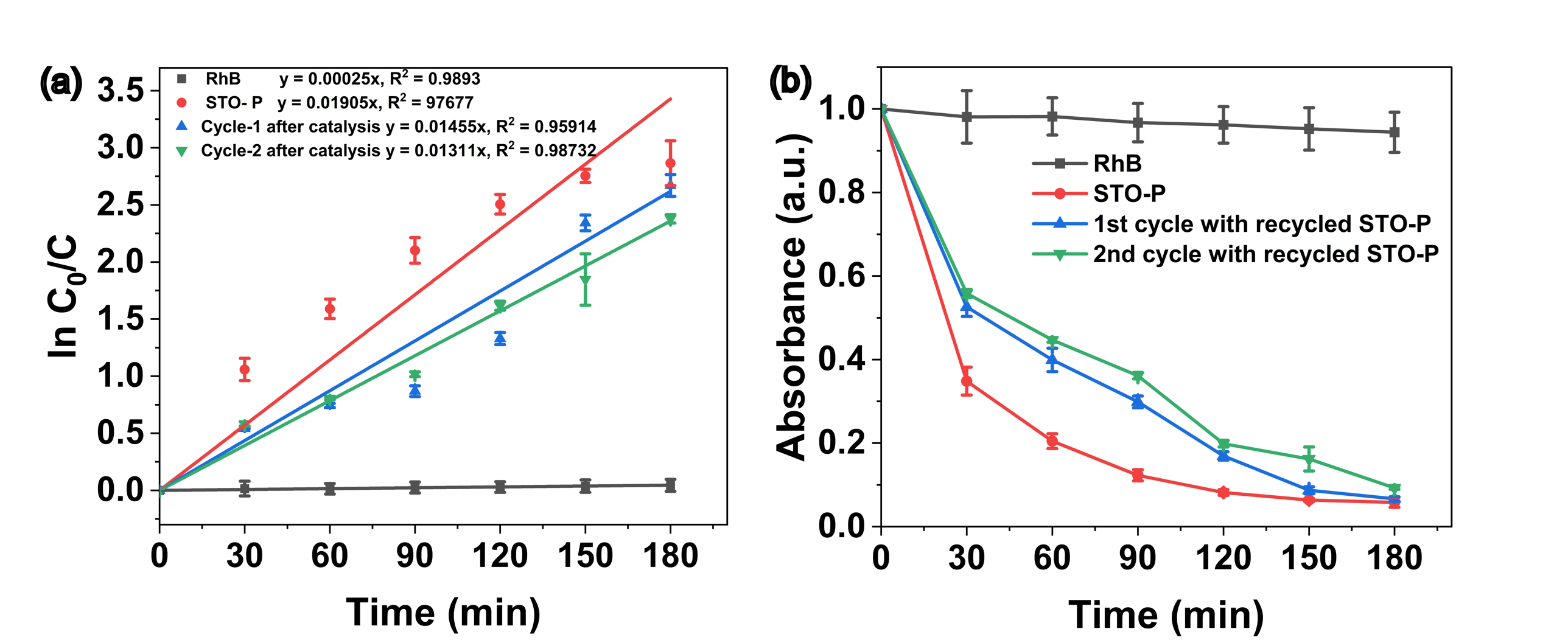 |
| --- |

**Figure S5: a)** Dye degradation kinetics of recycled STO-P sample and **b)** Overall dye degradation by recycled STO-P.

| 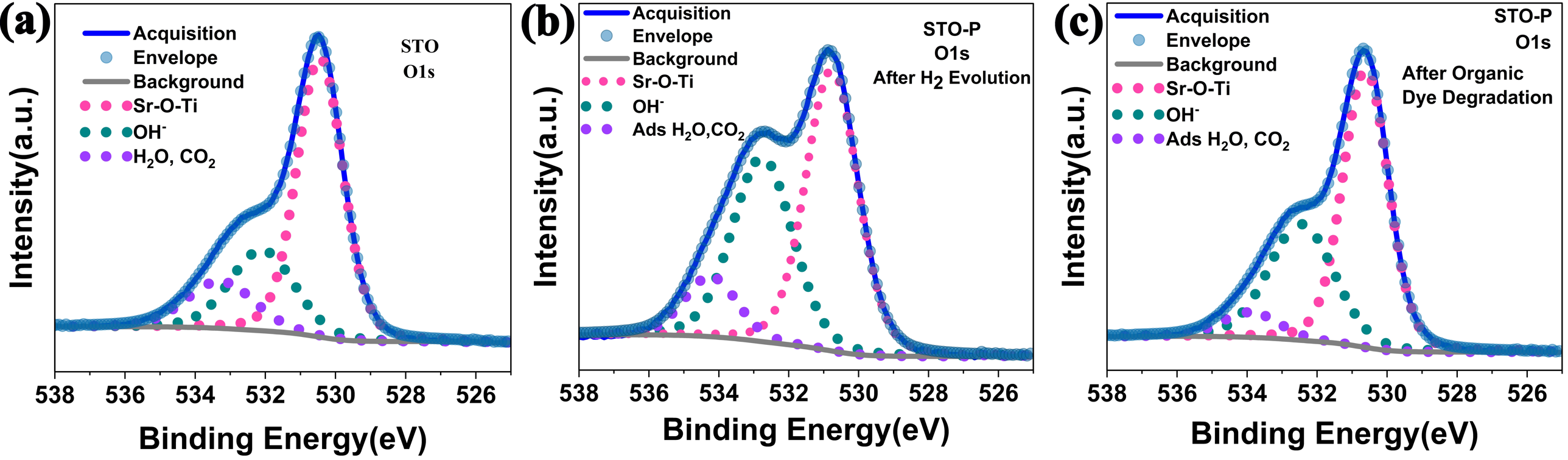 |
| --- |

**Figure S6:** **a)** XPS O1s spectrum with peak fitting before hydrogen evolution. **b)** XPS O1s spectrum after hydrogen evolution. **c)** XPS O1s spectrum with peak fitting after organic degradation.

| 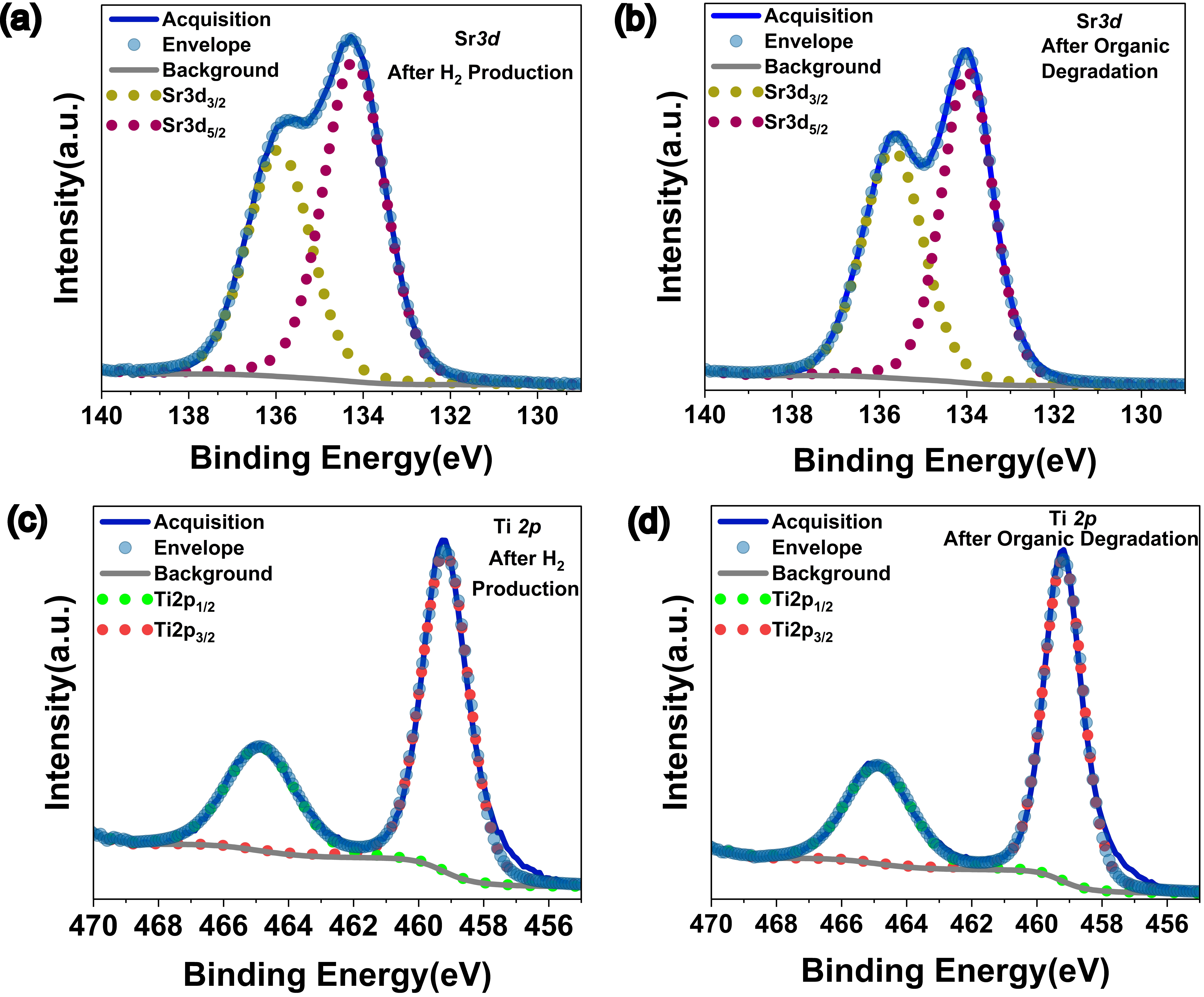 |
| --- |

**Figure S7:** **a)** Deconvolution of Sr3d_₅/₂_ and Sr3d_₃/₂_ Peaks in STO-P after hydrogen production. **b)** Deconvolution of Sr3d_₅/₂_ and Sr3d_₃/₂_ Peaks in STO-P after organic degradation. **c)** XPS Ti 2p spectrum of STO-P with peak fitting after hydrogen production. **d)** XPS Ti 2p spectrum of STO-P with peak fitting after organic degradation.

Fixed constraint (bottom)


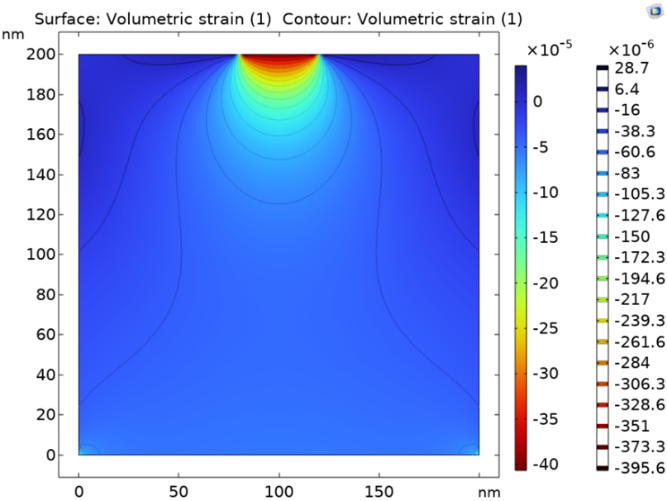


100 Mpa pressure

Affected area (40 nm in the center of the top) boundary)

Fixed constraint (bottom)

**Figure S8**: Simulated electric potential distribution in centrosymmetric STO nano-powder Induced by non-uniform mechanical strain.


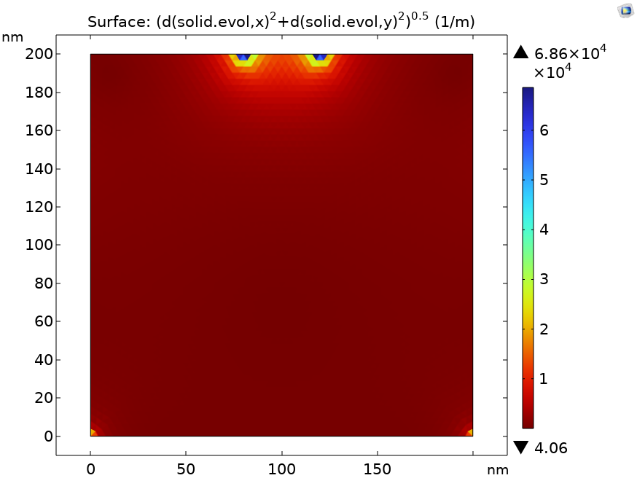


**Figure S9:** Stress-induced electric field distribution in STO nano-powder: Visualization of localized strain effects on flexoelectric polarization.
